# Design and fabrication of robust hybrid photonic crystal cavities

**DOI:** 10.1515/nanoph-2024-0500

**Published:** 2024-11-26

**Authors:** Alex Abulnaga, Sean Karg, Sounak Mukherjee, Adbhut Gupta, Kirk W. Baldwin, Loren N. Pfeiffer, Nathalie P. de Leon

**Affiliations:** Department of Electrical and Computer Engineering, 6740 Princeton University, Princeton, USA

**Keywords:** heterogeneous nanophotonics, III–V nanofabrication, quantum photonics

## Abstract

Heterogeneously integrated hybrid photonic crystal cavities enable strong light–matter interactions with solid state, optically addressable quantum memories. A key challenge to realizing high quality factor (*Q*) hybrid photonic crystals is the reduced index contrast on the substrate compared to suspended devices in air. This challenge is particularly acute for color centers in diamond because of diamond’s high refractive index, which leads to increased scattering loss into the substrate. Here, we develop a design methodology for hybrid photonic crystals utilizing a detailed understanding of substrate-mediated loss, which incorporates sensitivity to fabrication errors as a critical parameter. Using this methodology, we design robust, high-Q, GaAs-on-diamond photonic crystal cavities, and by optimizing our fabrication procedure, we experimentally realize cavities with *Q* approaching 30,000 at a resonance wavelength of 955 nm.

## 1 Introduction

Color centers and other atom-like defects in the solid state are a promising platform for long distance quantum networks because they can have long spin coherence times and efficient spin-photon interfaces, and they can be incorporated into scalable photonic devices [1], [2]. Some of the most sophisticated quantum network demonstrations to date are based on color centers in diamond such as the nitrogen-vacancy (NV) [3], [4], [5], [6] and silicon-vacancy (SiV) centers [7], [8], as well as rare earth ions in various host materials [9], [10], [11], [12], [13]. These qubits can be incorporated into nanophotonic cavities to enhance light–matter interactions and achieve improved spin-photon entanglement rates [14]. One approach to cavity integration is to mill [15], [16] or etch [17], [18], [19], [20] monolithic cavities out of the host material, but this can introduce surface defects and subsurface damage that can degrade the spin and optical properties of the qubit [21], [22], [23], [24], [25], [26]. An alternative approach is to fabricate the photonic device using another material that is well-suited for photonics fabrication and then couple the qubit to the evanescent field by placing the cavity on the substrate containing the qubit, allowing the qubit to reside in a more pristine environment. This strategy has been recently deployed for Er ions in various host materials [11], [27], [28], [29], [30], [31], [32], Yb ions in YVO_4_ [33], and SiV centers in diamond [34]. The key challenge for hybrid photonic crystal cavities is scattering into the high index substrate, which leads to lower *Q* as compared to devices in air. This is particularly challenging for material systems with a low index contrast and operation wavelengths in the visible range. While hybrid devices with *Q* up to 190,000 have been demonstrated for silicon (*n* = 3.5) on CaWO_4_ (*n* = 1.9) in the telecom band [11], for lower index contrast devices such as GaP-on-diamond [34], the demonstrated *Q* is limited to less than 10,000 in the visible wavelength range. Previous work with ultra-low index contrast platforms have achieved quality factors as high as 36,000 in the telecom band and 7,000 in the visible by encapsulating the cavity and extending the cavity geometry to overcome substrate scattering losses at the cost of much larger mode volume [35], [36].

The typical methodology for designing 1D photonic crystals is to maximize the expected Purcell factor by designing a structure with a mode volume (*V*) close to the minimum possible value, and then maximizing *Q*. This can be accomplished by maximizing the photonic band gap and lengthening the tapered region that defines the cavity mode and then iteratively optimizing the cavity parameters to achieve the highest *Q*/*V*. However, despite hybrid device designs that can achieve *Q* exceeding 1 million in simulation [33], [37], [38], these high Qs have not been experimentally realized in fabricated devices.

Here, we demonstrate that robustness to fabrication errors is a critical parameter in designing photonic crystal cavities that is independent of the designed *Q*. We perform random sampling of cavity designs under realistic fabrication errors and observe a significant scatter in the simulated error-sensitivity across designs. Armed with this design methodology, we then show that previously demonstrated hybrid photonic crystal cavities are likely limited by fabrication errors, not by sidewall roughness, absorption losses, or other scattering mechanisms. We focus on GaAs-on-diamond [37] as a model system that is suitable for coupling to neutral SiV centers in diamond [39]. In order to fabricate devices with the correct target parameters, we optimize the lithography, etching, and undercut chemistry to realize GaAs-on-diamond hybrid photonic crystals with *Q* approaching 30,000 at a resonance wavelength of 955 nm and exceeding 43,000 at 1,520 nm. We hypothesize that these demonstrations have now achieved material-absorption-limited *Q*.

## 2 Hybrid photonic crystal design

The key figure of merit in designing an optical cavity is the Purcell enhancement of the emitter spontaneous emission rate, given by $P=4g{\left(\vec{r}\right)}^{2}/\kappa \Gamma_{0}$ , where Γ_0_ is the native spontaneous emission rate of the optical transition of interest, $g\left(\vec{r}\right)$ is the single-photon Rabi frequency, and *κ* is the cavity decay rate. The Rabi frequency is determined by the overlap between the cavity electric field, $\vec{E}\left(\vec{r}\right)$ , and the emitter dipole moment, $\vec{\mu }$ , as $g\left(\vec{r}\right)=\vec{\mu }\cdot \vec{E}\left(\vec{r}\right)/\hbar$ . The cavity decay rate of a resonance at frequency *ω* is defined as *κ* = *ω*/*Q* where *Q* is the quality factor of the resonance. To maximize the Purcell factor, we seek cavity designs that achieve resonances with high *Q* and concentrated electric field at the emitter’s location. We then separately model the sensitivity of these designs to fabrication error and incorporate this robustness as design criteria.

**Figure 1:**
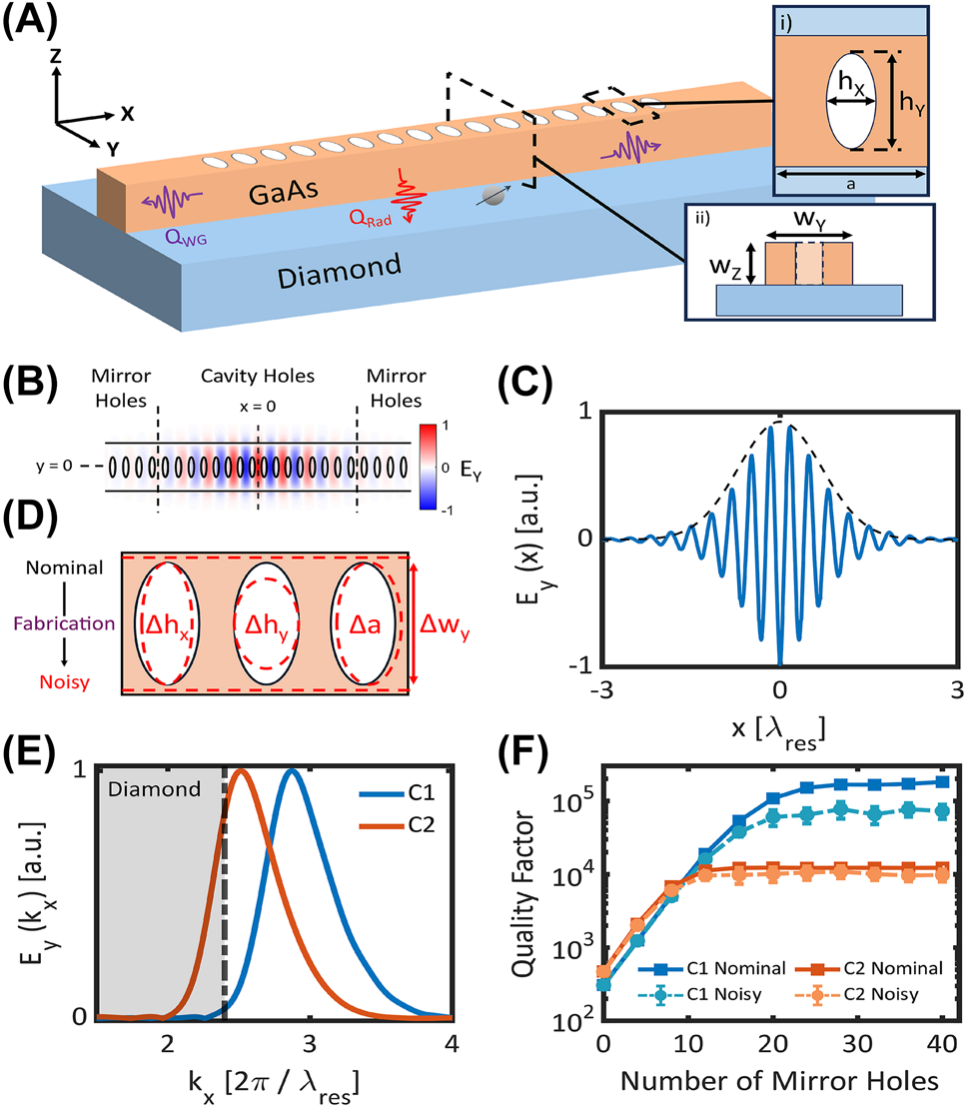
Overview of hybrid PhC design process. (A) Schematic of a hybrid GaAs-on-diamond photonic crystal cavity. The cavity is defined by a one-dimensional lattice of elliptical holes with minor diameter, *h*_*x*_, major diameter, *h*_*y*_, lattice constant, *a*, beam width, *w*_*y*_, and thickness, *w*_*z*_. Insets (i) top-down and (ii) cross-sectional views of a unit cell. (B) 2D mode profile of a resonance overlaid with the underlying cavity geometry. The mode is concentrated at the center of the cavity and decays toward the mirror region. (C) 1D slice of the mode profile taken at *y* = 0. The cavity profile decays from the defect with a Gaussian envelope due to the linear increase in mirror strength from unit cell to unit cell in the cavity region. (D) Schematic of fabrication-induced errors to the hole diameters (Δ*h*_*x*_, Δ*h*_*y*_) and placement (Δ*a*), and to the beam width (Δ*w*_*y*_) of the cavity. (E) 1D Fourier transform of the mode profiles of cavities *C*_1_ and *C*_2_. The shaded gray region indicates *k*_*x*_ vectors below the diamond light line. (F) Nominal and noisy Q-scaling of cavities *C*_1_ and *C*_2_. Each noisy data point consists of 30 independent simulations. Error bars indicate 95 % confidence intervals on the fit to the mean *Q*. Solid and dashed lines are guides to the eye.

We consider a one-dimensional GaAs-on-diamond photonic crystal cavity comprised of a periodic array of elliptical holes in a nanobeam waveguide as shown in Figure 1A. The cavity unit cell is parameterized by the hole diameters, (*h*_*x*_, *h*_*y*_), lattice constant, (*a*), and cross-sectional area, (*w*_*z*_, *w*_*y*_). In contrast to free-standing cavities, there are several challenges with designing hybrid photonic devices. The lack of *z*-symmetry complicates index guiding, and the reduced index contrast between the cavity and the substrate restricts the range of guided effective indices. Due to the high index of diamond in particular, previous designs required local etching of the diamond to achieve a large photonic bandgap [40]. More recently, we proposed a design procedure for achieving large band-gap unit cells without etching into the diamond by performing a grid-search over the parameter space using the periodic eigenmode solver MIT photonics bands (MPB) [37], [41].

Our approach begins by sweeping the lattice constant of a unit cell. As we are interested in modes at the *k*_*x*_ = *π*/*a* point in reciprocal space, we can relate the lattice constant to a target effective index, *n*_0_, as *a* = *λ*_0_/2*n*_0_ where *λ*_0_ is the target resonance wavelength. We consider a target wavelength *λ*_0_ = 955 nm, near the zero-phonon line of the neutral SiV center in diamond [39]. The target wavelength has been adjusted from 946 nm to account for a blue-shift of the cavity resonance at low temperatures due to the temperature dependence of the GaAs index of refraction [42]. The effective index of a guided mode must lie between the indices of diamond and GaAs, i.e., *n*_0_ ∈ (2.4, 3.5), which bounds the range of lattice constants. For each lattice constant in this range, we independently sweep the nanobeam cross-sectional parameters, *w*_*z*_ and *w*_*y*_, and for each cross section, we vary the hole diameters according to a single parameter, the fill factor, *f* ∈ (0, 1), such that $h_{x}=a\sqrt{f}$ and $h_{y}=w_{y}\sqrt{f}$ . Our approach results in four independent parameters that define the design space. The metric of interest is the unit cell mirror strength, defined as the separation between the target frequency and the nearest quasi-transverse-electric (TE) guided mode, normalized by the target frequency [S.1]. For a given unit cell, a mirror is formed by periodically arraying the cell in a one-dimensional lattice, and a cavity is created by introducing a defect in the lattice.

In order to understand the impact of fabrication errors on cavity *Q*, we must first understand the origin of losses in the ideal case. It has previously been shown that the quality factor of a cavity is determined by the degree of coupling between the cavity mode and radiative modes [43]. In a hybrid geometry, the high-index substrate greatly expands the light cone of radiative modes that can result in significant overlap between the cavity mode and leaky modes in the substrate. To achieve high-Q designs, we require a defect geometry that minimizes overlap between the cavity mode and the diamond leaky modes. We introduce a defect in the mirror lattice by quadratically chirping the lattice constant of the unit cell from the mirror periodicity, *a*_*mir*_, so as to pull the fundamental quasi-TE band to create a guided mode at the target frequency [S.1]. The lattice constant that matches the fundamental TE band to the target frequency is defined as the cavity periodicity, *a*_*cav*_. A quadratic chirp is chosen as it results in a linear change in mirror strength in the cavity region, yielding a Gaussian decay envelope as shown in Figure 1C and described in [44], [45]. The real-space Gaussian envelope corresponds to a Gaussian mode profile in reciprocal space, centered at a k-vector, *k*_*res*_ = 2*πn*_*res*_/*λ*_*res*_ where *n*_*res*_ is the resonance effective index and *λ*_*res*_ is the resonance wavelength. The adiabaticity of the chirp can be increased by using more cavity holes, resulting in higher *Q* at the expense of an expanded mode volume [S.2]. In this work, we consider designs with 16 cavity holes, resulting in mode volumes on the order of one cubic wavelength.

As an example of the relationship between the cavity mode profile and *Q*, the reciprocal mode profile of two cavities, *C*_1_ and *C*_2_, are plotted in Figure 1E, where (*a*_*mir*_, *a*_*cav*_, *w*_*z*_, *w*_*y*_, *h*_*x*_, *h*_*y*_) = (172, 156, 220, 430, 71, 186) nm for *C*_1_ and (184, 162, 220, 350, 78, 149) nm for *C*_2_. While both cavities have a similar shape to their mode profiles, *C*_2_ has a lower effective index, resulting in substantial overlap with radiative modes in the diamond substrate. To illustrate the impact this overlap has on the cavity quality factors, we perform finite-difference time-domain (FDTD, Lumerical) simulations of each cavity as a function of the number of mirror holes as shown in Figure 1F.

For a given cavity, the quality factor can be described as 1/*Q* = 1/*Q*_*wg*_ + 1/*Q*_*i*_ where *Q*_*wg*_ refers to leakage through the ends of the cavity and into the nanobeam waveguide and *Q*_*i*_ represents the intrinsic cavity quality factor. As the number of mirror holes is increased, decay into the nanobeam is reduced, and the cavity quality factor approaches its intrinsic value. The intrinsic quality factor of a cavity can be approximated by the saturated quality factor, *Q*_*sat*_. The number of mirror holes required to reach saturation varies between designs according to the unit cell mirror strength. By comparing the saturated quality factor of cavities *C*_1_ and *C*_2_, we observe an order of magnitude difference, consistent with the amount of spectral overlap with the leaky modes. As such, finding high-Q designs can be understood as finding designs with minimal overlap between the cavity mode and radiative modes, and thus finding designs with a high effective index for the cavity mode.

**Figure 2:**
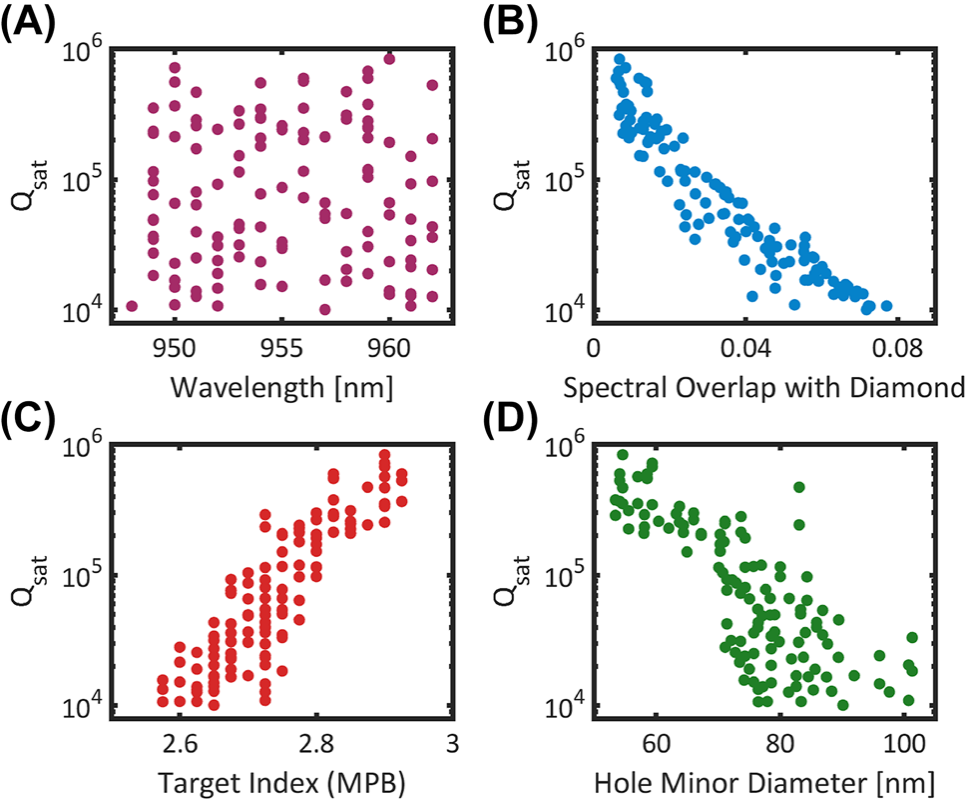
FDTD quality factor simulations of 200 randomly sampled cavities. Each cavity is sampled without bias from a set of 10,000 unit cells generated from MPB simulations. (A) Simulated *Q*_*sat*_ versus wavelength for different cavity designs near the target wavelength of 955 nm. To ensure saturation, the number of mirror holes is dynamically adjusted as a function of mirror strength for each cavity. (B) Saturated quality factors plotted against the cavity mode overlap with the diamond light line, (C) the unit cell target index, and (D) the hole minor diameter, *h*_*x*_.

To investigate the generality of this approach, we simulate a set of 10,000 unique unit cells with appreciable mirror strength using MPB. We randomly sample from these unit cells without bias, and for each unit cell, we construct a cavity by finding the appropriate *a*_*cav*_. For each cavity, we simulate *Q*_*sat*_ by dynamically adjusting the number of mirror holes for each design. The results for 200 such cavities near the target wavelength are shown in Figure 2A. We observe a range of Qs from 1.0 × 10^4^ to 8.4 × 10^5^. By analyzing the cavity profiles, we can compare the saturated quality factors to the amount of spectral overlap with the diamond radiative modes as shown in Figure 2B. The overlap quantity is calculated by taking the Fourier transform of a 2D slice of the cavity mode profile and computing the fraction of spectral components with *k*_*x*_ vectors that lie below the diamond light line. We observe a clear correlation between the saturated quality factors and the amount of spectral overlap. As we use an identical chirp function for all designs, the overlap primarily depends on the resonance mode effective index [S.3]. To achieve high *Q*, a high effective index is thus necessary. In Figure 2C, we compare the saturated quality factor of the sampled cavities with the unit cell target index used in the MPB sweep. As expected, we observe a clear correlation between the target effective index and the simulated quality factor. Furthermore, the mode volume can be minimized by selecting higher mirror strength designs that compress the cavity profile along the nanobeam axis [S.3]. As such, maximizing *Q*/*V* for a cavity can be simplified to maximizing the target index and mirror strength of the unit cell. By focusing on unit cell simulations, we can significantly reduce the computational overhead of the design process by deterministically screening for designs that achieve these criteria.

While these results present a clear roadmap to designing high *Q*/*V* photonic crystals, they do not account for the practical challenges of device fabrication, where quality factors often trail the design limit by orders of magnitude [20], [33]. The discrepancies between simulated and measured quality factors can arise from a variety of fabrication imperfections such as sidewall roughness or feature placement and sizing errors. In particular, the impact of lattice disorder on light confinement has long been studied in two-dimensional photonic crystal cavities and photonic crystal waveguides [46], [47], [48], [49]. To account for such errors in the design process, we develop a simple model for simulating fabrication imperfections in our cavity geometry.

We consider errors in the hole diameters, lattice constant, and beam width of the cavity as shown in Figure 1D. For a given cavity with some nominal saturated quality factor, *Q*_0_, we generate a noisy cavity instance by applying an error to every hole and to the entire beam width. For every hole and each parameter, the errors are independently sampled from identical normal distributions with standard deviations (*σ*_*hx*_, *σ*_*hy*_, *σ*_*a*_, *σ*_*wy*_), respectively.

The *Q* of the noisy cavity can then be directly simulated using FDTD. By repeatedly generating and simulating independent noisy cavity instances, we can extract a mean expected *Q*_*sat*_ for a given cavity under a specific amount of error. To accurately estimate the fabrication errors in our devices, we image fabricated devices with scanning electron microscopy (SEM) and develop an image processing algorithm to collect statistics on the hole sizes and variance of fabricated devices [S.4]. From this analysis, we calculate an upper bound on our errors as (*σ*_*hx*_, *σ*_*hy*_, *σ*_*wy*_) = (2 %, 2 %, 1 %). We estimate the lattice constant error as *σ*_*a*_ = 0.75 nm for our electron beam lithography tool.

**Figure 3:**
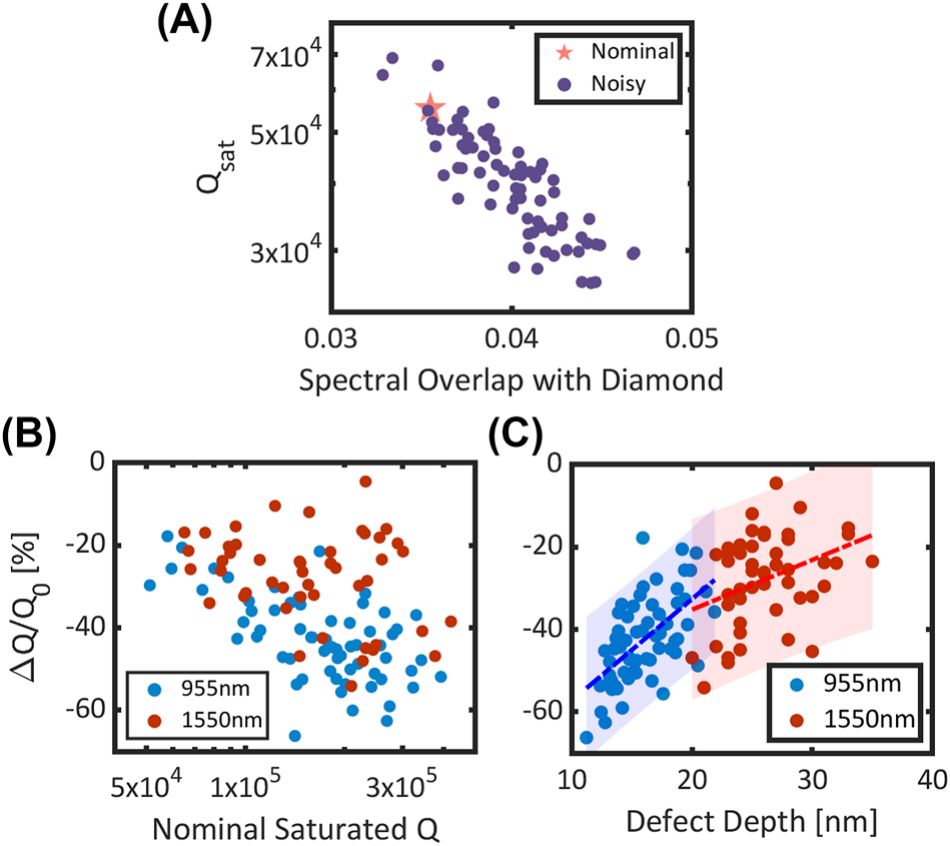
Fabrication error simulations. For a given cavity design, the error-sensitivity analysis is performed by repeatedly generating and simulating noisy cavity instances. The mean quality factor, (*Q*_*μ*_), of the noisy cavities is extracted and compared to the nominal quality factor, (*Q*_0_), to compute the relative change, (Δ*Q*/*Q*_0_). (A) Error-sensitivity analysis for cavity *C*_3_, with (*a*_*mir*_, *a*_*cav*_, *w*_*z*_, *w*_*y*_, *h*_*x*_, *h*_*y*_) = (174, 160, 220, 450, 83, 216) nm. For each noisy-cavity simulation, the saturated quality factor and spectral profile is extracted. The decrease in quality factor of the noisy cavities correlates with increased spectral overlap with leaky modes in the diamond. (B) Relative change in quality factor as a function of nominal quality factor for randomly sampled cavities at 955 nm and 1,550 nm. Each data point is the mean of 30 noisy simulation instances. (C) Relative change in quality factor as a function of cavity defect depth, defined as the change in period between the mirror and cavity region. The dashed blue line is a linear fit to the 955 nm data, and the shaded blue region indicates the 95 % confidence intervals of the fit. The root mean squared error of the fit is 8.35 %. The dashed red line is a linear fit to the 1,550 nm data, and the shaded red region indicates the 95 % confidence intervals of the fit. The root mean squared error of the fit is 10.46 %.

Figure 1F shows an example Q-scaling simulation for cavities *C*_1_ and *C*_2_ in the presence of these simulated errors. At low numbers of mirror holes, the nominal and noisy quality factors are similar, implying that the errors minimally affect the amount of light outcoupled to the nanobeam waveguide. Practically, this implies that the mirror strength is minimally perturbed by the errors. At larger numbers of mirror holes where *Q* approaches saturation, we observe a decrease in the noisy *Q* relative to the nominal *Q*. On average, the noisy cavities yield a lower intrinsic quality factor as compared to the nominal cavity. Analyzing the mode profiles of the noisy cavities at saturation, we observe that the cavities experience a perturbation to their mode profiles, which leads to increased overlap with radiative modes in the substrate as shown in Figure 3A.

Comparing three cavities, *C*_1,2,3_, we observe that each design experiences a different relative change in quality factor under the same noise distributions. This indicates that certain designs are more sensitive to fabrication errors than others. The sensitivity of a cavity to fabrication errors can be calculated by the relative change in the saturated quality factor, defined as Δ*Q*/*Q*_0_ = (*Q*_*μ*_ − *Q*_0_)/*Q*_0_ where *Q*_*μ*_ is the mean *Q* of the noisy cavity simulations. To identify designs that are more robust to errors, we randomly sample cavities from Figure 2A and perform the noise analysis. In addition, we repeat the analysis for cavities designed to operate in the telecom band. The results show a large spread in the relative sensitivity of different designs (Figure 3B). We observe that the telecom cavities can achieve higher robustness to fabrication errors, particularly at higher nominal *Q*, as compared to the 955 nm cavities. This result is consistent with prior examples in the literature where hybrid cavities in the telecom band have achieved much higher Qs than their visible wavelength counterparts [11], [29], [33], [34].

To understand the cause of these variations, we analyze the relative change in *Q* as a function of various cavity parameters [S.5]. While there are many parameters that may affect the design sensitivity, we observe a correlation between fabrication robustness and larger defect depths as shown in Figure 3C. The defect depth of a cavity is defined as the total change in periodicity from the mirror region to the cavity region (*a*_*mir*_ − *a*_*cav*_). This result indicates that under the applied errors, one particular cause of increased spectral overlap is likely due to errors in the placement of the holes. Cavities with larger defect depths will necessarily require larger steps in the lattice constant for the same number of cavity holes. As such, the fractional error in placement is smaller for these cavities, resulting in a smaller relative perturbation to the mode. Designs at longer wavelengths, i.e., larger feature sizes, will necessarily experience less of a perturbation. Using this model, designs can be screened by their fabrication robustness to maximize the realized quality factor. By correlating the robustness to a single parameter, the defect depth, we can reduce the sample space when performing the noise analysis.

## 3 Fabrication

**Figure 4:**
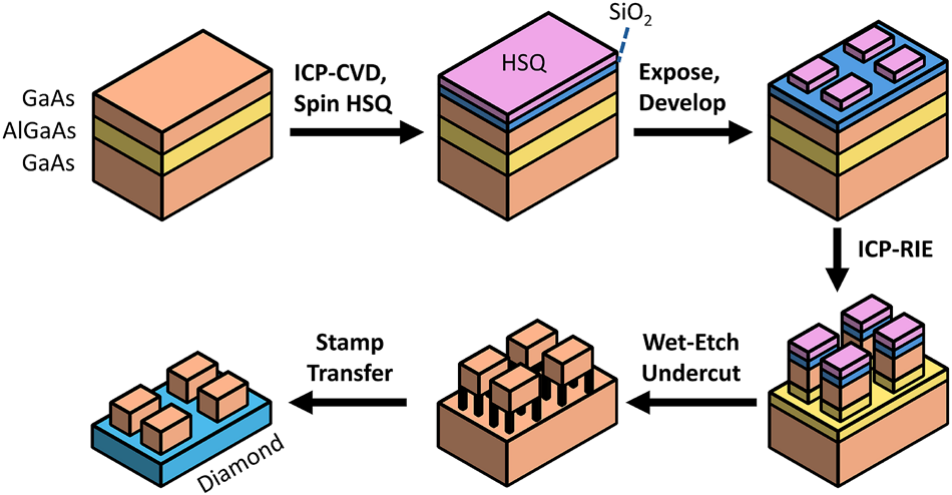
Fabrication flow diagram for GaAs-on-diamond photonic crystals. Beginning with an epitaxial GaAs-Al_0.8_Ga_0.2_As-GaAs wafer, we deposit a 5 nm SiO_2_ adhesion layer using an ICP-CVD, then spin HSQ, and expose at 100 KeV. Following development, patterns are transferred into the substrate with an ICP-RIE, then undercut and transferred to diamond for measurement.

We fabricate hybrid GaAs-on-diamond photonic crystals using a stamp-transfer approach [9], [34] in which devices are fabricated off-chip and then transferred onto the diamond as shown in Figure 4. Beginning with an epitaxial GaAs wafer, we pattern photonic crystals using electron-beam lithography (EBL) and transfer the patterns into the device layer using an inductively coupled plasma reactive ion etch (ICP-RIE). The devices are then released using a selective wet etch of a sacrificial AlGaAs layer and transferred onto diamond using a polydimethylsiloxane (PDMS) stamp. Full fabrication details can be found in [S.6].

To achieve high *Q*, we focus on designs with high effective indices. In general, high effective indices will correspond to designs with larger cross-sectional areas and smaller holes. For wavelengths in the visible or near-infrared (NIR), the hole sizes required can become prohibitively small. In Figure 2D, we plot the saturated quality factor of the 955 nm cavity designs as a function of the hole minor diameter, *h*_*x*_. We observe that for the highest *Q* designs, holes with *h*_*x*_ < 60 nm are required. Writing such small holes can be challenging due to limitations in EBL resist contrast and resolution. Furthermore, etching small holes or trenches incurs effects of aspect-ratio dependent etching (ARDE) [50]. While smaller features can be written by using thinner resist, this can lead to mask erosion and roughness during the etching process. This is further exacerbated by the ARDE phenomenon as longer etches are required to fully clear the small features.

In order to verify the accuracy of the fabrication sensitivity simulations of the previous section, it is critical that device performance is not limited by poor fabrication in the form of sidewall roughness, imperfect hole etching, or residue. As such, we develop a fabrication procedure for photonic crystals with small holes by optimizing the EBL, etching, and undercut processes. To write small holes in a device, it is necessary to use a high-contrast, high-resolution resist with excellent etch selectivity. We use hydrogen silsesquioxane (HSQ) as it exhibits high selectivity, which allows for thinner resists and thus smaller features. However, HSQ contrast is much lower than other common EBL resists such as polymethyl methacrylate (PMMA) or ZEP [51]. As such, there has been significant effort to maximize the HSQ contrast by manipulating the development conditions through postexposure baking [52] and developing at elevated temperatures [53].

**Figure 5:**
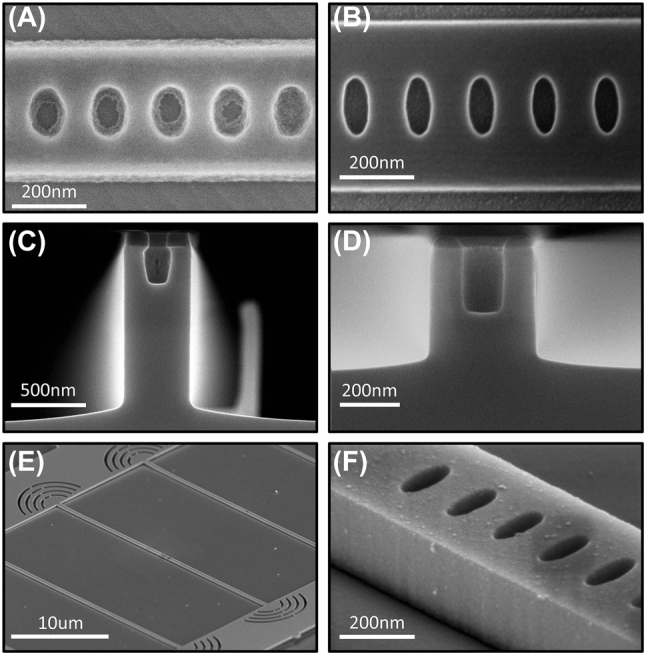
Optimized fabrication of high aspect ratio PhCs. (A) Example photonic crystal written in HSQ and developed using standard TMAH. The pattern was written at a dose of 650 μc/cm^2^ and developed in 25 % TMAH for 2 min. (B) Example photonic crystal written in HSQ and developed using salty-TMAH. Sample was written at a dose of 5,700 μc/cm^2^ and developed in 1 % NaCl in 25 % TMAH at 30 °C for 4 min. (C) Cross-sectional SEM image of a photonic crystal hole with (*h*_*x*_ = 70 nm, *h*_*y*_ = 130 nm) etched with a high-flow etch. The etch was performed with 10 sccm each of Cl_2_, BCl_3_, Ar_2_, and N_2_ at a pressure of 5mTorr, RF power of 50 W, and ICP of 500 W. Etch time was 30s. (D) Cross-sectional SEM image of the ARDE optimized etch. The etch was performed with 1.75 sccm Cl_2_, 1 sccm N_2_, and 2 sccm Ar at a pressure of 2mTorr, RF power of 25 W, and ICP of 50 W. Etch time was 330s. (E) SEM image of a device pattern after stamp transfer on diamond. The sample was coated with 3 nm of iridium to prevent charging during imaging. (F) Magnified image of the photonic crystal nanobeam. The observed residue on the cavity results from the iridium sputtering process for imaging.

In Figure 5A, we show an example of a section of a photonic crystal waveguide written in HSQ and developed in 25 % tetramethylammonium hydroxide (TMAH) in water. The sample is baked before and after development at 200 °C on a hotplate. For a given design, as the written dose increases, the holes approach the target dimensions before saturating. Further increasing the dose, or reducing the pattern diameters, results in undesired HSQ development within the holes. By manipulating the density of nearby features, the degree of overdosing within the holes can be reduced, indicating that electron scattering from nearby writing is a key factor limiting the hole contrast [S.6.1]. Under these development conditions, we observe a minimum hole diameter of *h*_*x*_ = 85 nm.

To write the holes necessary for the highest *Q* designs, we seek to further optimize the development process to improve the HSQ contrast without sacrificing resist thickness or pattern density. It has previously been observed that the addition of salts to alkali developers can significantly increase the contrast of HSQ by enhancing the dissolution rate, leading to a more aggressive development [54], [55]. The contrast has been shown to further be enhanced by optimizing the temperature of the solution during development [56]. While these results have primarily focused on maximizing the density and size of lines, we expect the increased contrast to aid in mitigating the effects of electron scattering in the holes of our photonic crystals. We prepare a mixture of 1 % NaCl in 25 % TMAH, heated to 30 °C, hereto referred to as “salty TMAH.” In comparison to standard TMAH development (*γ* = 2.03), we observe a significant increase in contrast (*γ* = 3.83) for the salty TMAH [S.6.1]. The saturated dose increases from 1,150 μC/cm^2^ for standard TMAH to 4,900 μC/cm^2^ for salty TMAH. In Figure 5B, we show an example of a pattern developed in salty TMAH. The mask achieves significantly higher contrast and reduced line-edge roughness, and minor diameters as small as 55 nm can be written with a high density of nearby writing. One trade-off with the salty development is the high dose required, which significantly increases the required write time. However, higher doses allow for improved etch selectivity as the HSQ matrix density is increased by the longer exposure [57].

While these results allow for writing small holes, an additional challenge is to transfer the patterns into the device layer via etching. In a single-step etch process, pattern transfer is achieved through a combination of simultaneous physical etching, chemical etching, and passivation. As an example, in Figure 5C, we show a cross-sectional SEM image of a typical hole in a photonic crystal device. The pattern was etched using a combination of Cl_2_, Ar, BCl_3_, and N_2_ gases at high flow rates and high power. After etching, the chip was scribed and manually cleaved before mounting vertically within an SEM to image the cross section. In this etch, the chlorine radicals generated in the plasma react with the GaAs substrate through a chemical process. The chemical reaction between the chlorine and GaAs is isotropic in nature, and so to achieve vertical sidewalls high RF powers are used to direct ICP radicals. This, however, results in significant physical bombardment of the mask, reducing the achievable selectivity. To overcome this challenge, passivating gases are used to help achieve anisotropic etching with more moderate RF powers [58], [59], [60]. In particular, N_2_ has been shown to be an excellent passivating agent for Cl_2_-based etching of GaAs [61], [62]. High flow rates are typically used for the chemical gases to achieve rapid etch rates in the substrate while minimizing mask erosion. By tuning these parameters, it is possible to achieve extremely high selectivity and vertical sidewalls in macroscopic regions.

However, etching inside the small holes of the photonic crystal introduces several challenges. The etch rate is severely reduced inside the hole and also exhibits a high degree of isotropy, leading to a “bottling” effect. The reduction of the etch rate as a function of feature size is known as RIE-lag [50] and is a form of ARDE. ARDE is a phenomenon in which the etch rate within a hole or trench decreases as the aspect ratio increases. The aspect ratio is defined as the etch depth divided by the width of the opening. As the etch depth increases, the aspect ratio also increases, further reducing the etch rate and leading to a self-limiting effect [63]. As a result, using thicker masks effectively increases the aspect ratio of the structure, and thus ARDE cannot be overcome by simply using thicker masks with longer etch times. The microscopic origin of ARDE can be explained by neutral shadowing [64], while the increased isotropy can be explained by radical scattering within the holes [65].

To overcome these challenges, we develop an etch recipe optimized for hole-based photonic crystal cavities by simultaneously minimizing ARDE, maximizing selectivity, and minimizing sidewall roughness. To mitigate the effects of radical scattering, we seek to minimize the overall density of radicals within the plasma. We begin by reducing the gas flow rates to the minimum supported by the tool and reduce the ICP power to the minimum able to support a stable inductively coupled plasma. To improve selectivity, we reduce physical bombardment of the mask by reducing the RF power to the lowest stable value. The pressure of the chamber is reduced to the lowest supported value to reduce sidewall roughness, while the Ar flow is adjusted to stabilize the plasma. Finally, the relative ratios of Cl_2_ and N_2_ are adjusted to deterministically control the sidewall angle of the holes and beam to achieve vertical profiles [S.6.2]. The resulting etch is shown in Figure 5D and demonstrates a significant reduction in ARDE while maintaining vertical sidewalls.

Following etching, the sacrificial layer is partially removed using hydrochloric acid, and the HSQ is stripped using dilute hydrofluoric acid. The undercut parameters are selected to fully suspend the cavities while mitigating residue [S.6.3]. After the undercut, the devices are transferred onto diamond using a PDMS stamp. In Figure 5F, we show an example device after stamp-transfer onto diamond. The resulting devices exhibit smooth, vertical sidewalls and minimal residue.

## 4 Measurement

To verify our fabrication error modeling, we fabricate several different cavity designs with a similar nominal quality factor, but with a range of different simulated sensitivities to fabrication errors. Figure 5E shows an example of a pattern of devices on diamond. A device consists of a single cavity with grating couplers on both ends, and each pattern consists of seven identical cavities. For a given cavity design, we sweep the number of mirror holes across different patterns. The entire set of device patterns is then repeated with shifts to the hole sizes and beam widths to account for write-to-write variations, resulting in 210 devices for each cavity design.

**Figure 6:**
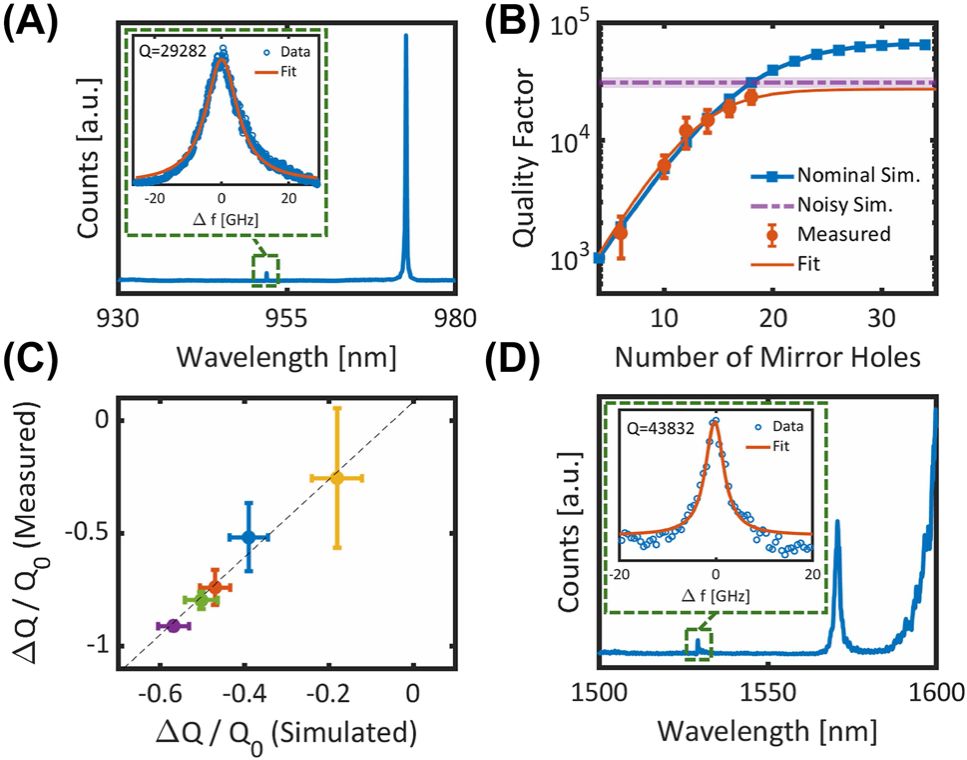
Measurement results of fabricated cavities. (A) Transmission spectrum of a typical cavity. The resonance at 952 nm is the fundamental mode, while the feature at 975 nm is a higher-order mode. Inset: Lorentzian fit of the resonance from which the quality factor is extracted. (B) Q-scaling measurements of a cavity design compared to simulation. The blue data points are the simulated Q-scaling of the cavity in the absence of fabrication errors. The blue solid line is a guide to the eye. The purple dashed curve is the mean-saturated quality factor of 70 noisy cavity simulations, and the shaded purple region represents 95 % confidence intervals on the fit to the mean. Red data points are the mean and standard deviation of the measured quality factors, and the solid red line is a fit to the measurement data. (C) Relative change in quality factor of five fabricated cavity designs compared to simulation. The dashed line is a linear fit to the data. X-error bars are 95 % confidence intervals on the fit to the mean noisy-Q. Y-error bars are 95 % confidence intervals on the fitted intrinsic *Q*. (D) Example transmission measurement of a cavity in the telecom C-band.

Transmission measurements of the cavities are performed using a free-space confocal microscopy setup [S.7]. Figure 6A shows the transmission spectrum of a typical cavity. By measuring cavities at different numbers of mirror holes, we can fit the intrinsic quality factor of the fabricated cavity. The measurements are fit to a model of 1/*Q* = 1/*Q*_*wg*_ + 1/*Q*_*i*_ with *Q*_*wg*_ = *Ae* ^*bM*^ where *M* is the number of mirror holes. The fitting parameters (*A*, *b*, *Q*_*i*_) are left as free parameters without bounds. In physical terms, *A* ≡ *Q*(*M* = 0) is the quality factor of the cavity at zero mirror holes. The parameter *b* is related to the mirror strength of the design and dictates the rate at which the cavity reaches saturation. Figure 6B shows a typical Q-scaling fit of a cavity design, showing good agreement between the simulated saturated *Q* in the presence of fabrication error, and the fitted intrinsic *Q*.

We repeat this process for five different cavity designs with different simulated robustness. The full measurements can be found in [S.8]. By comparing the fitted intrinsic *Q* to the predicted noisy saturated *Q*, we find excellent agreement in the relative sensitivities to fabrication error of the different designs. These results indicate that e-beam placement and feature size errors are indeed the dominant loss mechanism for the cavities. Additionally, we observe that the fabricated devices demonstrate a higher degree of sensitivity to errors than the simulated values, further illustrating the importance of selecting robust designs.

To illustrate the generality of our design and fabrication process, we fabricate photonic crystals designed for the telecom C-band. In Figure 6D, we demonstrate an example transmission measurement of a cavity with a resonance near 1,520 nm with quality factor approaching 45,000. This result is comparable to recent demonstrations of hybrid silicon photonic crystal cavities with comparable index contrast [9], [29], [30], [31].

## 5 Conclusions

In this work, we demonstrate that robustness to fabrication errors is a critical design parameter for hybrid photonic crystal cavities that can be directly modeled in FDTD. The simulated robustness varies across designs, independent of the nominal quality factor, which is the typical parameter that is optimized in the design process.

We show that the nominal *Q* is determined by the degree of coupling between the cavity mode and substrate leaky modes and can be maximized by selecting high effective index unit cells. By correlating the nominal *Q*, mode volume, and robustness to unit cell parameters, we are able to screen unit cells based on their potential to maximize *Q*/*V* and fabrication robustness. This result significantly reduces the computational burden of the design process, as specific unit cells can be deterministically selected for simulation rather than relying on random sampling. As the unit cell simulations can be performed rapidly in comparison to full cavity FDTD simulations, we can probe a significant portion of the design space with reduced computational overhead. Additionally, optimization techniques could be used to create unit cells that simultaneously achieve the desired attributes.

Using our fabrication sensitivity model, we can search for designs, which simultaneously maximize the nominal quality factor while minimizing fabrication error sensitivity. We fabricate cavities with target *Q*_0_ > 1 × 10^5^ at 955 nm, but we observe a departure from the model in fabricated devices at high *Q* around 30,000, independent of simulated robustness or fabrication processes [S.9]. There are many factors that could limit the device quality factor, including scattering losses related to fabrication or material absorption. As we observe a similar limit at both 955 nm and 1,520 nm, we can exclude fabrication-related scattering as this would scale as *λ* ^−4^. Similarly, multiphoton absorption in the material would be expected to scale more sharply with wavelength. As such, we hypothesize that the observed *Q* arises from material absorption due to bulk or surface defects. In particular, we observe that using commercial metalorganic chemical vapor deposition (MOCVD) GaAs wafers, the device quality factors are limited to *Q* below 10,000 at 955 nm [S.10]. By instead using high purity molecular-beam epitaxy (MBE) wafers, we observe an immediate threefold increase in quality factor for the high-Q designs. Owing to the ultra-clean vacuum environment and novel chamber design, these films have achieved record mobility with implied bulk defects below 1 part in 10 billion [66], [67], [68]. As such, we conclude that the improvement in device *Q* from the MOCVD to MBE wafers is likely due to a reduction in bulk defects.

One potential source of loss may be mid-gap states in the surface oxide, specifically along the device sidewalls. Previous work has shown that surface passivation techniques can dramatically improve device *Q* in GaAs photonic devices [69], [70], [71], [72], [73]. Alternatively, it has also been shown that oxygen segregation during growth of the sacrificial AlGaAs layer can create impurities that propagate into the device layer [74]. As we use a large sacrificial layer thickness for device suspension and transfer (see S.6.3), the quantity of oxygen-segregated defects may be significant in our device layer. To further improve device *Q*, modifications to the wafer stack or the addition of passivation steps may be required. Despite these limitations, the demonstrated cavity quality factors at 955 nm represent the highest experimentally realized *Q* for hybrid cavities in the visible or NIR to the best of our knowledge, while our results at 1,520 nm are comparable to the state of the art for silicon cavities with similar index contrast. These results were enabled by improvements in the design procedure, as well as through optimization of the fabrication processes.

Owing to the generality of the design process, our model could be readily applied to arbitrary wavelengths and material stacks. By separating device fabrication from the substrate, the hybrid photonics platform could be utilized to significantly expand the space of candidate qubits for quantum network experiments. The full design code used in this work is available at github/deLeonPhotonics.

## Supplementary Material

Supplementary Material Details
