# Supplementary material for: Design and fabrication of robust hybrid photonic crystal cavities
Source: Nanophotonics. 2024 Nov 26;14(11):1927–37. doi: 10.1515/nanoph-2024-0500 (PMC12133251; doi:10.1515/nanoph-2024-0500)
Supplement: Supplementary file 1 — Supplementary Material Details [file j_nanoph-2024-0500_suppl_001.pdf]

Supplementary Materials for

Design and Fabrication of Robust Hybrid Photonic Crystal Cavities

Alex Abulnaga[1,†], Sean Karg[1,†], Sounak Mukherjee[1], Adbhut Gupta[1], Kirk W. Baldwin[1], Loren Pfeiffer[1], and Nathalie P. de Leon[1,*]

[1]Department of Electrical and Computer Engineering, Princeton University, Princeton, USA

[†] These authors contributed equally to this work

[*]corresponding author, email: npdeleon@princeton.edu

S.1 Cavity Design and Photonic Band Diagrams

In figure S.1 we outline the procedure for designing the one-dimensional photonic crystal cavities discussed in this work. As discussed in the main text and in a prior work [1], we begin by analyzing single unit cells consisting of an elliptical hole in a nanobeam waveguide. Using MIT photonics bands (MPB) [2], we simulate the frequency bands of a unit cell at a given point in reciprocal space. The metric of interest is the mirror strength, defined according to equation 1, where f_0, f_{TE0} and f_{TE1} are the target frequency and frequencies of the first two quasi TE bands at the $k_x = \pi/a$ point in reciprocal space.

$$\text{Mirror Strength} \equiv \frac{\min\left[(\mathrm{f_{TE1}} - \mathrm{f_0}), (\mathrm{f_0} - \mathrm{f_{TE0}})\right]}{\mathrm{f_0}} \tag{1}$$

By varying the cross-sectional area, periodicity, and fill factor, we can perform a grid search of possible unit cells. In figure S.1B we plot the full quasi TE band diagram of the mirror unit-cell for cavity C_1 from the main text. For the emitter to couple to the cavity, it is is necessary to shift the bands such that a guided mode exists at the target frequency. This can be achieved by modifying the periodicity of the unit-cell while keeping all other parameters fixed. As shown in figure S.1C, reducing the periodicity of the hole shifts the quasi TE modes to higher frequencies. The number of cavity holes and the functional form of the chirp from the mirror region to the cavity region dictates the adiabaticity of the introduced defect mode. We use a quadratic chirp function where the periodicity of a given unit-cell in the cavity region is given by

$$a(i) = a_{cav} + \frac{(a_{mir} - a_{cav})}{N^2} i^2 \tag{2}$$

where $N = \frac{N_{cav}}{2} - 1$ and $i \in (0, 1, 2, ..., N)$. N_{cav} is the total number of cavity holes used for the chirp. The functional form is chosen so that the first hole in the cavity region is at the cavity periodicity, i.e. $a(0) = a_{cav}$, and the last hole is at the mirror periodicity, $a(N) = a_{mir}$ as shown in figure S.1A.

S.2 Cavity Chirp Adiabaticity

To understand the impact of the number of cavity holes in the chirp region, we consider the example of cavity C_1. We simulate the saturated quality factor (Q_{sat}) and mode profile of the cavity as a function of the number of cavity holes as shown in figure S.2. As the number of cavity holes is increased, Q_{sat} increases, but so does the mode-volume. As the metric of interest is Q/V, choosing the exact number of cavity holes can depend on the Q-factors achievable in practice for a given material and fabrication process. The increase in both mode volume and quality factor can be explained by studying the cavity mode profiles. As an example, in figure S.2B we compare the real-space mode profiles along the nanobeam axis for cavity C_1 with 12 and 20 cavity holes. We observe that for 12 cavity holes, the mode-profile decays more rapidly along the nanobeam axis. As we are using fewer holes to chirp from a_{cav} to a_{mir}, the mirror-strength is increased in larger steps as compared to 20 cavity holes. This narrowing of the mode-profile in real-space necessarily corresponds to a broadening in reciprocal space, and thus greater spectral overlap with diamond leaky modes as shown in figure S.2C.

S.3 Additional Unit Cell Simulation Results

In figure 2 of the main-text, the Q_{sat} of 200 cavity designs and their mode-profiles are analyzed. We observe that the saturated quality factor depends on the amount of spectral overlap with leaky modes in the diamond. As we use an identical quadratic chirp function for all cavities, we expect that the amount of spectral overlap will primarily depend on the effective index of the cavity resonance. In figure S.3A we plot the amount of spectral overlap between the cavity mode and the diamond leaky modes as a function of the MPB target index. We observe a clear anti-correlation between the target indices and the spectral overlap. As such, higher target indices result in less overlap. However, we observe a trade-off in mode-volume as shown in figure S.3B. Despite the apparent trade-off, the ratio of Q/V for the cavities still scales with the target index, illustrating the importance of using high effective index designs. Additionally, we observe that the mode-volume of the cavity depends on the unit-cell mirror strength as shown in figure S.3C. Designs with larger mirror-strength will necessarily yield a more compressed mode-profile along the nanobeam axis. This points to two parameters, n_0 and mirror-strength, that can be optimized simultaneously at a unit-cell level to achieve high Q/V cavities.

S.4 Fabrication-Error Analysis

To estimate the amount of error to use in our robustness simulations, we develop an image-processing algorithm to directly analyze scanning electron microscope (SEM) images of our fabricated devices. In figure S.4 we show the results of an example fabrication run and subsequent analysis. Following electron beam

lithography, etching, and undercutting of the cavity design (see section S.6), we acquire SEM images of the holes in the the fabricated cavities. For a given number of cavities across the chip, we image every hole in the nanobeam. The algorithm uses the openCV package [3] to identify the hole contours and fit them to ellipses as shown in figure S.4A. From this fit, we extract the minor and major diameters of the holes. In figures S.4B and S.4C we show the results for an example cavity. By analyzing the distribution across the holes for different designs, we estimate an upper bound on the hole errors.

S.5 Additional Fabrication Robustness Correlations

To understand the design-to-design variations in robustness, we analyze the simulated reduction in quality factor as a function of different cavity parameters for the 955 nm and 1550 nm cavities as shown in figure S.5. We observe that the robustness to the simulated errors primarily correlates with the defect depth of the cavities as shown in figure S.5A. This poses a trade-off however as the defect depth of a cavity appears to anti-correlate with the effective index as shown in figure S.5F, which in turn leads to an increase in sensitivity with higher effective indices as shown in figure S.5C. Achieving designs with high effective indices and large defect depths can allow for simultaneously high nominal Q and low sensitivity to error.

S.6 Additional Fabrication Details

To fabricate the hybrid GaAs-on-diamond photonic crystal cavities studied in this work, we utilize a stamp transfer process in which the devices are fabricated off chip before transferring onto the diamond as shown in figure 4 of the main text. We begin with an epitaxial GaAs-$Al_{0.8}Ga_{0.2}As$-GaAs wafer stack where the top GaAs layer is grown to our target device layer thickness, and the $2um$ thick $Al_{0.8}Ga_{0.2}As$ layer acts as a sacrificial layer. For the designs fabricated in this work, we use a device thickness of $w_z = 220$nm. Following solvent cleaning of the chip, we deposit a 5 nm SiO_2 adhesion layer at 250°C using an ICP-CVD (Oxford PlasmaPro 100). We then spin a layer of hydrogen silsesquioxane (HSQ) e-beam resist (Dischem H-SiQ 4%) at 6000 rpm to yield an 80 nm thick resist layer. The HSQ is stored in liquid nitrogen, and is allowed to thaw for 45 minutes before spinning. After spinning, the sample is dehydration baked at 80°C for 4 minutes prior to exposure. The patterns are then written into the resist layer at 100KeV (Raith EBPG5150+). The nanobeams are written using a 200 pA, 200 um aperture beam (2 nm spot size) at a dose of 5700 uC/cm^2, while the frames and gratings are written with a 20 nA, 300 um aperture beam (13 nm spot size) at a dose of 6500 uC/cm^2. A larger beam is used for the frames to reduce write time, while the smaller beam used for the cavities allows for higher resolution. The salty-TMAH developer is prepared by mixing 0.2g of

NaCl in 20mL TMAH (25% in water). The mixture is sonicated for 10 minutes to fully dissolve, then heated to 30°C on a hotplate. The device chip is baked on a hotplate at 200°C for 2 minutes, then transferred into the salty TMAH mixture for four minutes to develop with gentle agitation. The chip is placed into successive water beakers followed by IPA, then blow-dried with N_2 before a final post-development bake at 200°C for four minutes.

S.6.1 Improving HSQ-Contrast using Salty-TMAH

The addition of salt to the TMAH developer is critical for improving the resist contrast and mitigating HSQ development within the cavity holes. In figure S.6, we illustrate the impact of secondary electron scatter on the writing of a nanobeam. As we use a stamp transfer fabrication approach, the nanobeams are contained within a frame structure which is used for suspending and later transferring the devices. Despite the large distance between the nanobeam and the frame, and the use of proximity error correction, secondary electron scatter from the frame write results in significant overdosing within the nanobeam holes, setting a lower bound on the achievable hole diameters at h_x = 85nm. By removing the frame, holes with minor diameters as small as h_x = 70nm can be written. As such, elongating the nanobeams within the frames can mitigate the impact of the frame write, but this comes at the expense of reduced pattern density and device stability during the stamp transfer process. As such, we seek to improve the achievable resolution in the presence of secondary scatter from the surrounding features.

Previous work has shown that the addition of salt to TMAH can significantly improve the resist contrast by modifying the dissolution rate [4]–[6]. To verify these results, we analyze the resist contrast as a function of development condition as shown in figure S.7. To construct the thickness-dose curves, we pattern 20 um x 20 um squares of HSQ at different doses, then measure the resulting thickness using a profilometer (KLA-Tencor P15). The measured data can be fit according to the following equation [7]

$$t(E) = t_0 - \Delta t_{max} exp(-E/E_n^*) \tag{3}$$

where $t(E)$ is the resist thickness after development at a given dose E, t_0 is the saturated resist thickness, Δt_{max} is the maximum resist thickness post development, and E_n^* is a resist sensitivity term. By fitting the measured resist thickness data to this model, we can extract the resist contrast, γ, as

$$\gamma \equiv -ln(t_0/\Delta t_{max}) \tag{4}$$

We observe that the addition of salt increases the resist contrast, and raising the temperature of development further increases the contrast. Additionally, we observe similar results using an alternative HSQ source, Dow-Corning XR-1541-006.

S.6.2 ARDE-Optimized Etching

To transfer the patterns into the GaAs device layer, we develop an etch recipe optimized for mitigating the effects of aspect ratio dependent etching (ARDE). As discussed in the main text, ARDE is a phenomenon in which the etch rate within a structure depends on its aspect-ratio, defined as the depth of the structure divided by the width. To develop an ARDE-optimized recipe, we begin with a high-flow, rapid etch as described in table 1. The rapid etch rate achieves high selectivity and vertical sidewalls outside of the PhC holes but the etch rate is severely reduced inside the hole and suffers a high degree of isotropic etching as shown in figure 5 of the main text. To mitigate these effects, we focus on minimizing the etch-rate by reducing ICP power, pressure, and gas flow rates to the lower limits supported by the ICP-RIE tool (Plasma-Therm Takachi). The ARDE-optimized etch is described in table 2.

The presence of Cl_2 acts to chemically etch the GaAs with a high degree of isotropy, while N_2 acts as a passivating agent to prevent isotropic etching. As the minimum flow rate of our tool is 1 sccm for Cl_2 and N_2, we use this as a starting point for the etch. As shown in figure S.8A, the resulting etch profile demonstrates a significant reduction in ARDE as compared to the fast etch, but is overly passivated as indicated by the outwardly sloped sidewalls within the hole. To achieve vertical sidewalls, we seek to reduce the passivation. As we are at the lower limit, we can not reduce N_2 and thus must increase Cl_2 to adjust the balance between etching and passivation. Increasing the Cl_2 flow rate to 1.75 sccm results in vertical sidewalls within and outside the hole, but the increased etch rate results in slightly higher ARDE as shown in figure S.8B. Further increasing the amount of Cl_2 leads to a significantly faster etch-rate, more ARDE, and increased isotropy as shown in figure S.8C. By fine-tuning the relative concentrations, we can achieve vertical sidewalls within the PhC holes for a given design. Importantly, the degree of ARDE varies according to the size of the hole as shown in figures S.8D - S.8F, and so the etch-recipe must be optimized according to the smallest hole being fabricated.

S.6.3 Undercutting and stamp transfer

Following the dry etch, the samples are immediately transferred into IPA to prevent oxidation of the exposed $Al_{0.8}Ga_{0.2}As$ layer. The patterns are then undercut prior to stamping. While both hydrochloric acid (HCl) and hydrofluoric acid (HF) can be used to etch the sacrificial layer [8], we observe that using HF results in significant residue around the devices as shown in figure S.9A. In a previous work, the residue was believed to be organic in nature, stemming from polymerization of the e-beam resist during ICP-RIE [9]. However, the observed residue is not removed by solvent cleaning, oxygen descum, additional acid soaks, or base soak. The residue can be removed by digital etching techniques [10], but we observe this to result in unpredictable expansion of the PhC holes and narrowing of the nanobeam. The residue occurs for both ZEP and HSQ e-beam resists, and is not observed after etching prior to undercutting. Al-

ternatively, HCl does not produce any residue when undercutting the sacrificial layer as shown in figure S.9B. This confirms that the residue is specific to some reaction between the HF and sacrificial layer. When using HCl to undercut the devices, HF must eventually be used to remove the HSQ, which results in a new form of residue as shown in figure S.9C. However, as the HCl has removed the majority of the AlGaAs, the residue following HSQ stripping is not likely to be related to the HF undercut issue. EDS measurements confirm the residue to be organic, and soaking the chip in solvents can remove the residue, resulting in pristine devices as shown in figure S.10B. The solvent-cleaning procedure consists of soaking in Microchem Remover PG (Kayaku) at 80°C for 24 hours, followed by a 3 hour soak in acetone at 50°C. Following the solvent cleaning, the sample is transferred to IPA then dried using a critical point dryer (Baltec-030).

Finally, the cavities are transferred to diamond using a stamp transfer process as shown in figure S.10A and described in [11]. Using the stamp transfer technique, we transfer device areas as large as 2mm x 2mm, corresponding to up 4000 individual cavities. Prior to stamp transfer, the diamond surface is cleaned in a 2:1 piranha mixture and dehydration baked at 100°C for 10 minutes.

S.7 Measurement Setup

The cavities in this work are measured using a confocal microscopy setup as shown in figure S.11. The excitation channel consists of a tunable laser (Toptica CTL-950) steered with a 2-axis scanning galvo mirror (Thorlabs GVS012) through a 4f relay system. The beam passes through a linear polarizer and motorized half-wave plate into a 0.85 NA objective (Olympus LCPLN100XIR). The polarization optics allow for selective measurement of the cavity TE spectrum. To measure the transmitted light through the cavity, the detection channel is independently steered using an additional galvo mirror, and is combined with the excitation through the 4f system using a 50:50 cube beamsplitter. A silicon APD is used in conjunction with a voltage controlled attenuator for detecting counts. To image the devices under the objective, an additional 850 nm diode laser is included in the detection path which allows for confocal imaging across the objective field of view. From this confocal image, the individual devices and gratings can be identified, and control software is used to automatically track the in and out-coupling spots on the gratings, and to sweep the laser wavelength. For measurement of high-Q devices, a scanning Fabry-Perot interferometer (Thorlabs SA30-95) is used to accurately calibrate the resonance linewidths. For measurements in the telecom band, the excitation laser is replaced with a Santec TSL-770, and the silicon APD is replaced with a superconducting nanowire single-photon detector (SNSPD).

S.8 Additional Cavity Measurements

In figure S.12 we show the Q-scaling measurements of the five cavities plotted in figure 6 of the main text. For each cavity, we fit the measured data to find the intrinsic quality factor of the cavity. The fitted intrinsic Q is compared to the nominal and noisy simulations to benchmark the accuracy of the fabrication-error simulations.

S.9 Measurement Results of High-Q Designs

Given the success of the noisy-simulation model, we attempt to apply our design rules to cavities with higher nominal Q-factors. We select cavity designs with a range of simulated sensitivity to fabrication errors, but with quality factors in excess of 100,000. In measuring the cavities however, we observe a breakdown of the fabrication-error model as all designs saturate at quality factors of 30,000. The origin of this saturation can arise from either material losses or fabrication-induced losses.

S.10 Measurement Results using MOCVD GaAs Wafers

In figure S.14 we plot the measurement results for cavities fabricated using a commercial MOCVD GaAs wafer. In contrast to the MBE wafers used in the main text, we observe much lower quality factors. For the MOCVD wafers, the maximum achievable quality factors are below 10,000.

S.11 Purcell Enhancement of SiV^0

In this section we analyze the expected Purcell enhancement that can be achieved using the cavity designs discussed in this work. We consider the case of a SiV^0 emitter located at a depth of 50 nm in the diamond, consistent with the analysis in [1]. As discussed in the main text, the expected Purcell enhancement can be calculated as $P = 4g(\vec{r})^2/\kappa\Gamma_0$, where Γ_0 is the native spontaneous emission rate of the optical transition of interest, $g(\vec{r})$ is the single-photon Rabi frequency, and κ is the cavity decay rate. The Rabi frequency is determined by the overlap between the cavity electric field, $\vec{E}(\vec{r})$, and the emitter dipole-moment, $\vec{\mu}$, as $g(\vec{r}) = \vec{\mu} \cdot \vec{E}(\vec{r})/\hbar$. The cavity decay rate of a resonance at frequency ω is defined as $\kappa = \omega/Q$ where Q is the quality factor of the resonance. After taking into account the local-field correction of the spontaneous emission rate [12], the dipole moment of the SiV^0 center ($\vec{\mu}$) can be calculated as:

$$\Gamma_0 = \frac{1}{\beta} \cdot \left(\frac{3n_{Dia}^2}{2n_{Dia}^2 + 1}\right)^2 n_{Dia} \cdot \frac{|\vec{\mu}|^2\omega^3}{3\pi\epsilon_0\hbar c^3} \tag{5}$$

where β describes the fraction of the decay rate caused by spontaneous emission of the transition coupled to the cavity. Assuming that the SiV^0 center is a perfect two-level atom, and that $\beta = 1$, we can calculate the SiV^0 dipole moment as $|\vec{\mu}| = 6.03 \times 10^{-29}$ C-m where we have used the bulk spontaneous emission rate of SiV^0 centers $\Gamma_0 = 2\pi \times 88$ MHz [13]. Assuming that the SiV^0 dipole moment is oriented parallel to the cavity electric field polarization, the expected Purcell factor can be estimated. Importantly, the Purcell factor will depend on the location of the emitter with respect to the spatial cavity mode. As an example, we assume that the SiV^0 centres are located at a depth of 50 nm below the diamond surface. In figure S.15, we analyze the mode volume and the cavity field strength at a depth of 50 nm for the cavities simulated in figure 2 of the main text. To account for both the cavity mode volume and the field strength at the target depth, we compute an effective cavity mode volume, defined as $V_{eff} = V/|E(z_0)|$ where $|E(z_0)|$ is the maximum normalized field strength at the target depth $z_0 = 50$ nm.

As we consider a fixed number of cavity holes (16), and a fixed device layer thickness ($w_z = 220$ nm), we observe minimal variation in the mode volume and field strength at the target depth as compared to the variation in quality factors. As such, the expected Purcell enhancement primarily scales with the cavity quality factors [Fig. S.16]. As a corollary, the Purcell enhancement is thus greater for cavities with less spectral overlap between the cavity mode and radiative modes in the substrate [Fig. S.16D], despite the apparent increase of effective mode volume [Fig. S.16C].

References

[1] D. Huang, A. Abulnaga, S. Welinski, M. Raha, J. D. Thompson, and N. P. De Leon, "Hybrid III-v diamond photonic platform for quantum nodes based on neutral silicon vacancy centers in diamond," *Optics Express*, vol. 29, no. 6, p. 9174, 2021. DOI: 10.1364/OE.418081.

[2] S. Johnson and J. Joannopoulos, "Block-iterative frequency-domain methods for maxwell's equations in a planewave basis," *Optics Express*, vol. 8, no. 3, p. 173, 2001. DOI: 10.1364/OE.8.000173.

[3] G. Bradski, *The OpenCV library*, 2000.

[4] J. K. W. Yang and K. K. Berggren, "Using high-contrast salty development of hydrogen silsesquioxane for sub-10-nm half-pitch lithography," *Journal of Vacuum Science & Technology B: Microelectronics and Nanometer Structures Processing, Measurement, and Phenomena*, vol. 25, no. 6, pp. 2025–2029, 2007. DOI: 10.1116/1.2801881.

[5] J. Kim *et al.*, "Understanding the base development mechanism of hydrogen silsesquioxane," *Journal of Vacuum Science & Technology B: Microelectronics and Nanometer Structures Processing, Measurement, and Phenomena*, vol. 27, no. 6, pp. 2628–2634, 2009. DOI: 10.1116/1.3250261.

[6] M. Yan, J. Lee, B. Ofuonye, S. Choi, J. H. Jang, and I. Adesida, "Effects of salty-developer temperature on electron-beam-exposed hydrogen silsesquioxane resist for ultradense pattern transfer," *Journal of Vacuum Science & Technology B, Nanotechnology and Microelectronics: Materials, Processing, Measurement, and Phenomena*, vol. 28, no. 6, C6S23–C6S27, 2010. DOI: 10.1116/1.3504497.

[7] C. A. Mack, S. Jug, and D. A. Legband, "Data analysis for photolithography," presented at the Microlithography '99, B. Singh, Ed., Santa Clara, CA, 1999, p. 415. DOI: 10.1117/12.350829.

[8] C.-W. Cheng, K.-T. Shiu, N. Li, S.-J. Han, L. Shi, and D. K. Sadana, "Epitaxial lift-off process for gallium arsenide substrate reuse and flexible electronics," *Nature Communications*, vol. 4, no. 1, p. 1577, 2013. DOI: 10.1038/ncomms2583.

[9] G. Kirsanské, "Electrical control of excitons in semiconductor nanostructures: From quantum dots in photonic-crystal devices to the exciton mott transition in coupled quantum wells," Ph.D. dissertation, University of Copenhagen, Faculty of Science, Niels Bohr Institute, 2016.

[10] D. Huang, *Building Quantum Network Nodes Based on Neutral Silicon Vacancy Centers in Diamond*. Princeton University, 2021.

[11] A. M. Dibos, M. Raha, C. M. Phenicie, and J. D. Thompson, "Atomic source of single photons in the telecom band," *Physical Review Letters*, vol. 120, no. 24, p. 243 601, 2018. DOI: 10.1103/PhysRevLett.120.243601.

[12] H. T. Dung, S. Y. Buhmann, and D.-G. Welsch, "Local-field correction to the spontaneous decay rate of atoms embedded in bodies of finite size," *Physical Review A*, vol. 74, no. 2, p. 023 803, 2006. DOI: 10.1103/PhysRevA.74.023803.

[13] B. C. Rose *et al.*, "Observation of an environmentally insensitive solid-state spin defect in diamond," *Science*, vol. 361, no. 6397, pp. 60–63, 2018. DOI: 10.1126/science.aao0290.

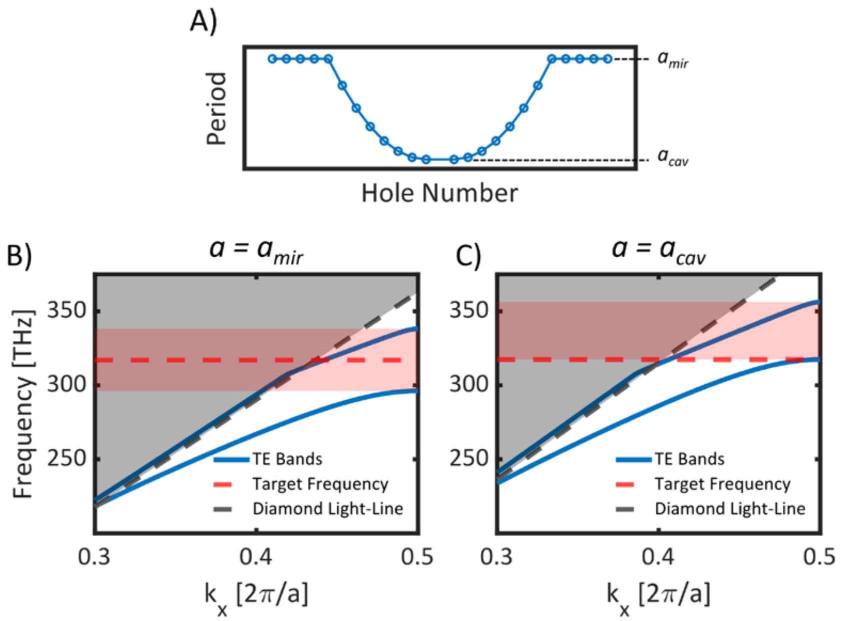

Figure S.1: Cavity construction and band diagrams. A) Cavity periodicity as a function of hole number. The period of the cavity holes are chirped from unit cell to unit cell using a quadratic taper function. B) Example band diagram of a mirror hole for cavitiy C_1. The shaded red-region indicates the band-gap for quasi TE modes C) Example band diagram at the center of the cavity. The quasi TE bands are pulled to higher frequencies by decreasing the period until the fundamental band matches the target frequency.

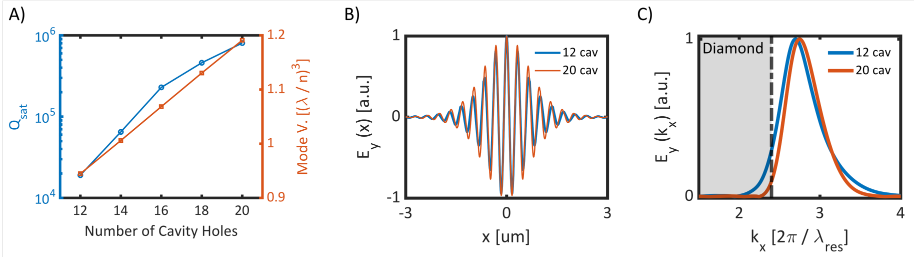

Figure S.2: A) Saturated quality factor and mode-volume of cavity C_1 as a function of the number of cavity holes. B) Real-space mode-profile of the cavity mode with 12 and 20 cavity holes. C) Fourier transform of the real-space mode profiles normalized by the simulated resonance wavelength.

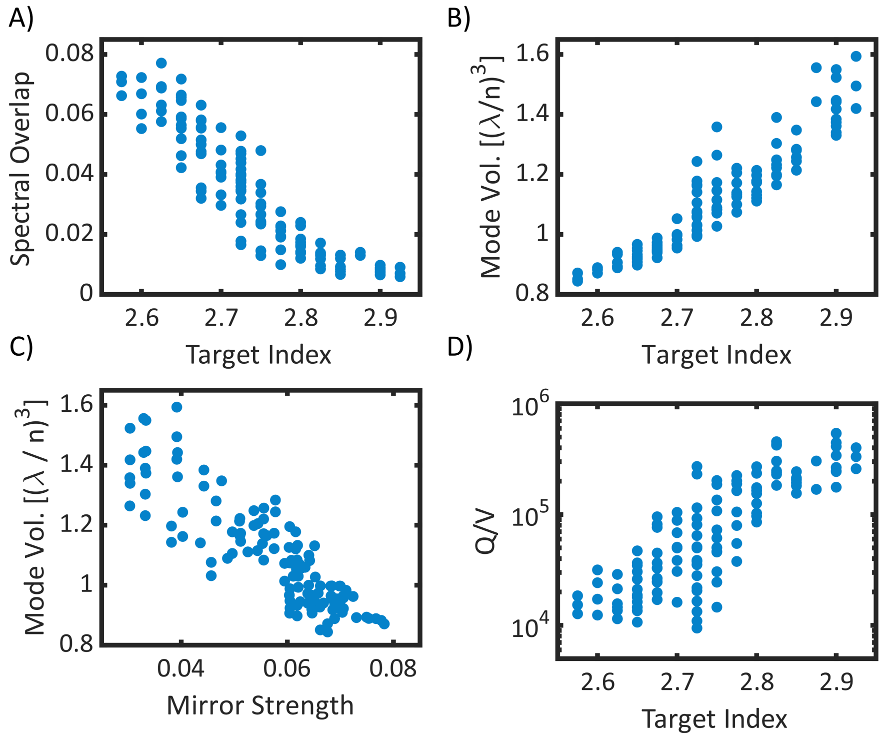

Figure S.3: A) Spectral overlap between the cavity mode-profiles and the diamond leaky-modes plotted as a function of the MPB target index. B) Cavity mode volume as a function of the target-index. C) Cavity mode volume as a function of the unit-cell mirror strength. D) Q/V as a function of target index.

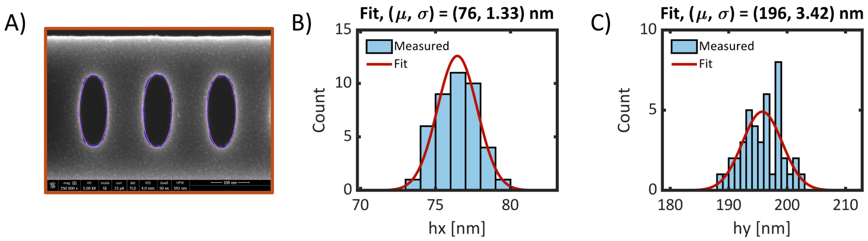

Figure S.4: A) Example SEM image of the holes in a fabricated cavity. The blue data-points are the identified contour edges, and the purple ellipses indicate the fit to the contours. Images are taken at 5KeV and 25pA at a magnification of 350,000x (Thermo-Scientific Verios-460 XHR SEM). B) h_x and C) h_y fit results of the holes in the device.

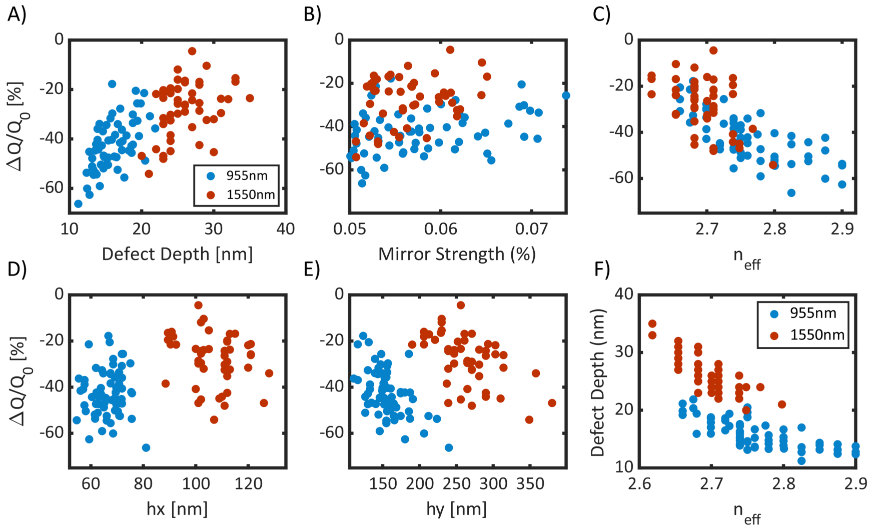

Figure S.5: Fabrication error sensitivity as a function of different cavity parameters. Simulated reduction in quality factor as a function of A) defect depth, B) mirror strength, C) target index, D) hole minor diameter, and E) hole major-diameter. F) Defect depth as a function of target index.

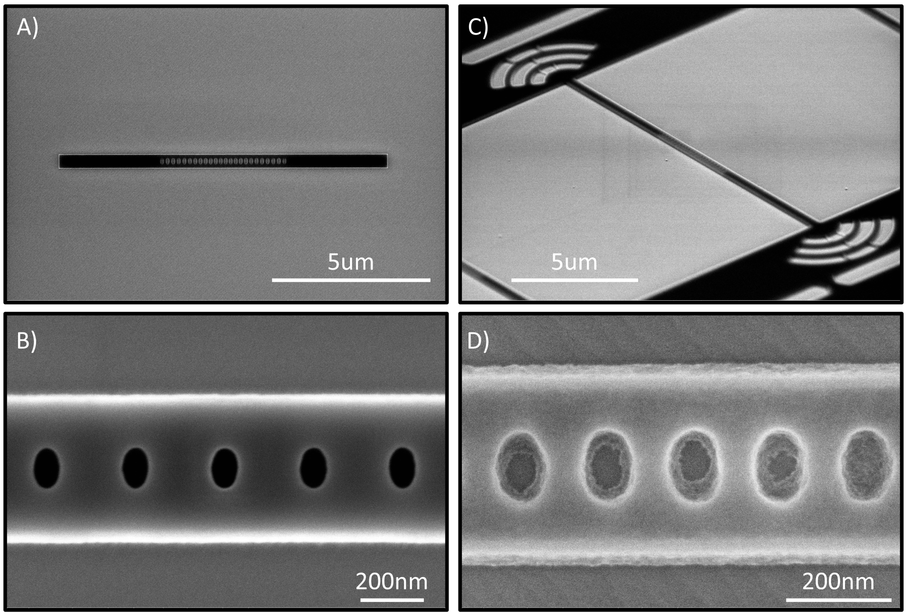

Figure S.6: Impact of frame-writing on cavity hole overdosing for devices developed using standard TMAH. A) SEM image of an isolated PhC waveguide pattern written in HSQ on GaAs. B) Zoom-in image of the isolated PhC waveguide, demonstrating holes as small as $(h_x, h_y) = (70nm, 130nm)$. C) A nanobeam written with the surrounding gratings and frame-structures necessary for suspending devices prior to stamp transfer. The PhC waveguide is 10um long. D) Zoom-in image of the PhC waveguide, illustrating overdosing within the PhC holes due to electron scatter from the frame write.

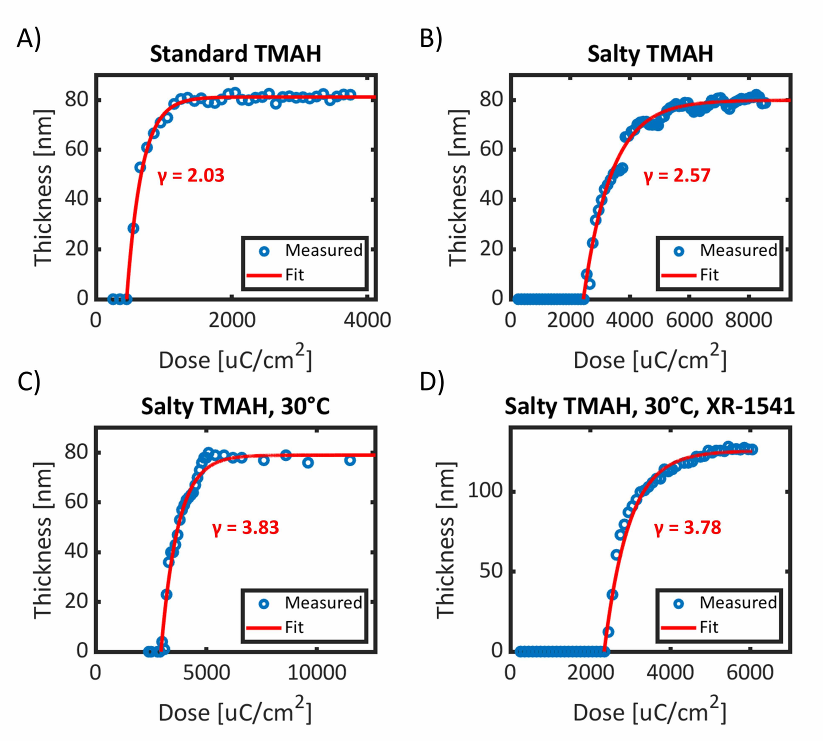

Figure S.7: Measured contrast curves for Dischem H-SiQ 4% developed with A) standard TMAH, B) room-temperature salty-TMAH, and C) heated salty-TMAH. D) Contrast curve for Dow-Corning XR-1541-006 HSQ developed in heated salty-TMAH. The measured data are fit to the model described in equation 3 and the contrast is extracted according to equation 4.

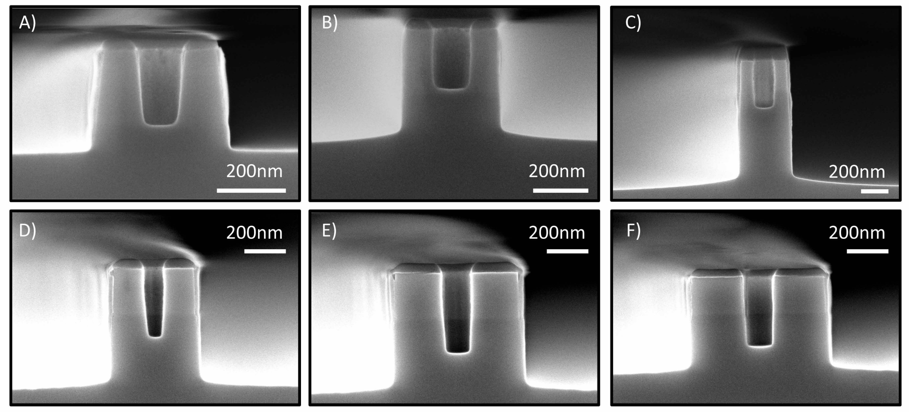

Figure S.8: ARDE etch recipe optimization A) Cross-sectional SEM of a PhC hole with h_x = 70nm and h_y = 130nm using the etch parameters in table 2 but with 1sccm of Cl_2. The etch is overly passivated, resulting in angled sidewalls. B) By increasing the Cl_2 concentration to 1.75 sccm, we achieve vertical sidewalls within the hole. C) Further increasing Cl_2 to 3sccm results in higher ARDE and increased isotropic etching. D) Cross-sectional SEM of circular holes with diameters of 100nm, E) 150nm, F) 200nm etched with the ARDE-optimized recipe. Reducing the hole-diameter exacerbates the ARDE and increases the degree of isotropic etching within the holes.

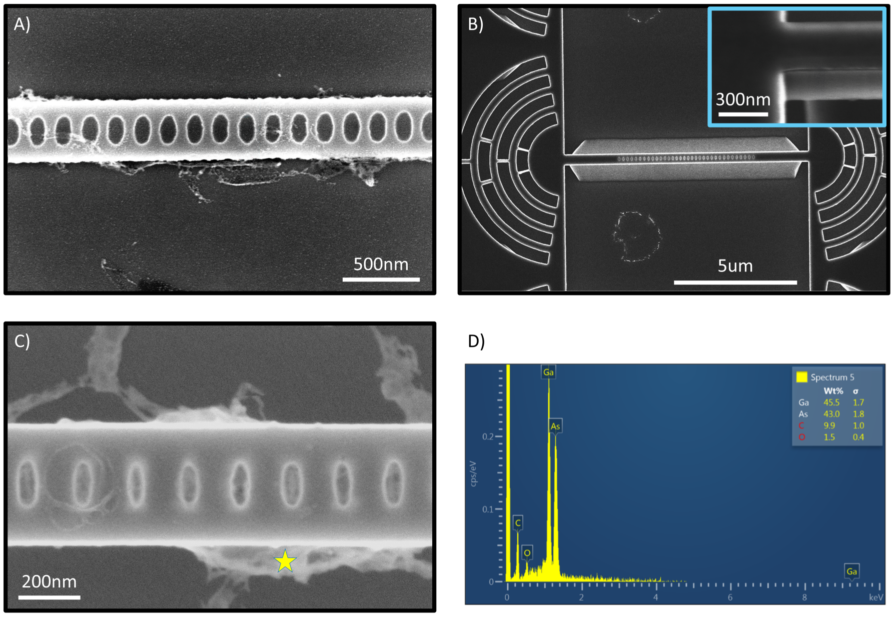

Figure S.9: A) Residue observed about the PhC after undercutting in HF. Sample was undercut in 1:4 (HF : DI water) for 65s. B) Device undercut with concentrated HCl, with no observable residue about the devices. C) Device undercut in 1:2 (HCl : DI water) for 8 minutes, followed by a dilute 1:15 (HF : DI water) HSQ-strip for 60s. The HCl undercut is performed in an ice-bath to improve selectivity of the etch. D) EDS spectra of the residue obvserved after HCl undercut and dilute HF HSQ-strip. The residue is organic in nature, and can be removed with solvent cleaning.

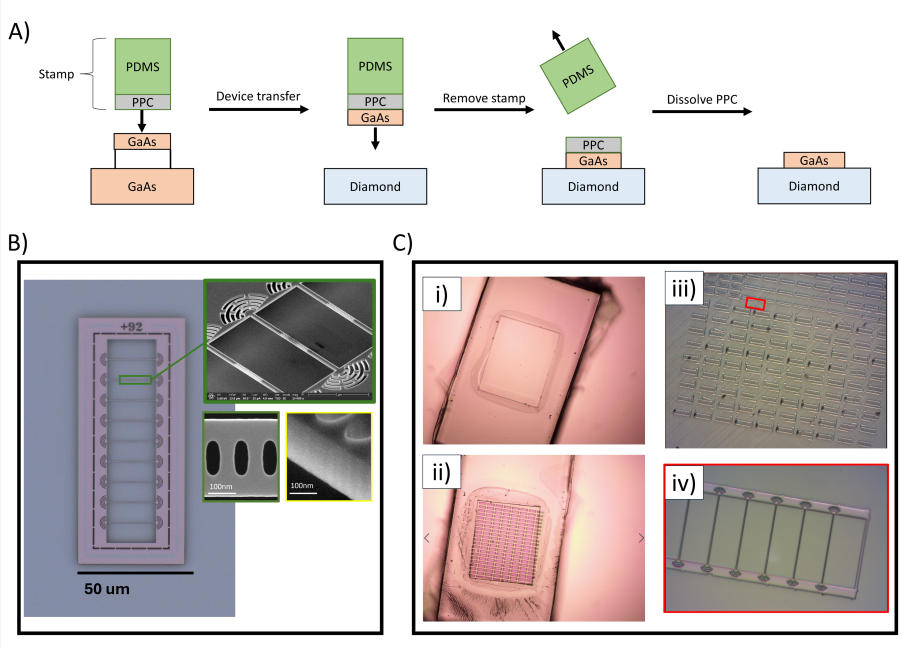

Figure S.10: A) Overview of the stamp transfer technique for placing GaAs PhCs onto diamond. B) Optical microscope image of a set of devices after undercutting. A given frame supports 7 to 11 identical devices. The shaded pink regions are free-standing, while the thin ring of gray about the outer frame represents the remnant AlGaAs which supports the structure. Insets show typical SEM images of the devices prior to stamp transfer, showing no residue after solvent cleaning. C) Stamp transfer process. Inset i) shows an example PDMS stamp with a PPC film over-top. The protudring rectangular region is the mesa which defines the area of device pick-up. ii) Optical image of a set of device patterns after pick-up with the stamp. iii) Devices on diamond after stamping and dissolving of the PPC film. iv) Example image of an in-tact frame of devices on diamond.

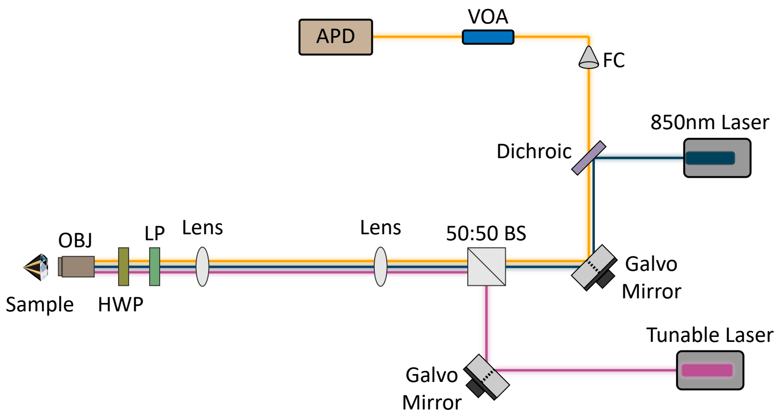

Figure S.11: Optical measurement setup used for measuring cavity spectra. Measurements are performed using a free-space 4f confocal measurement setup in conjunction with control software for beam steering and laser control.

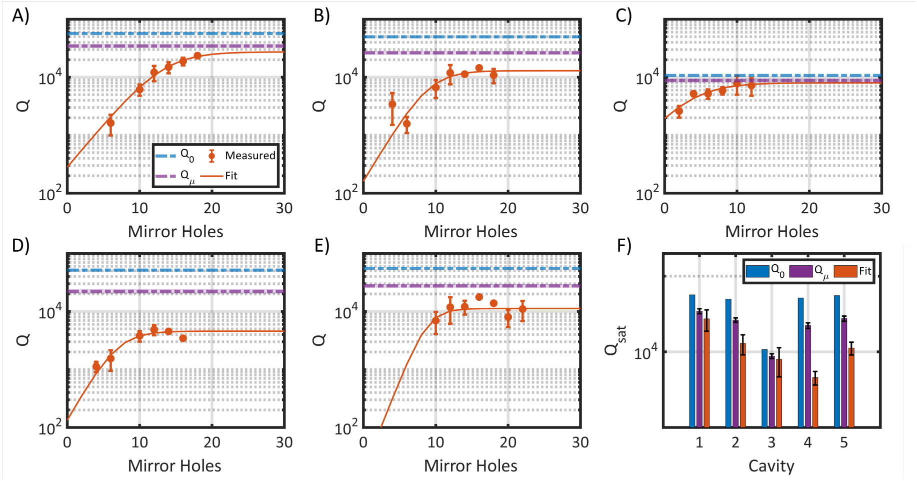

Figure S.12: Q-scaling measurements of the five cavities used to verify the fabrication-error simulation model. A) - E) Measured data plotted against the nominal and noisy simulations for five different cavity designs. F) Comparison between the nominal simulated Q-factor, noisy simulated Q-factor, and fitted intrinsic Q.

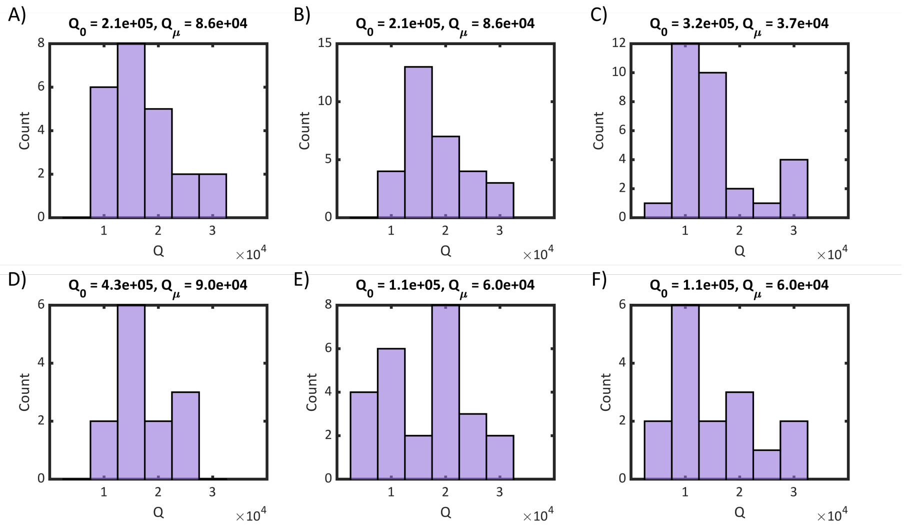

Figure S.13: Measurement results of several high-Q designs showing saturation at Q=30,000. A)-F) Measurement results of six different cavity designs with a range of simulated nominal Q and fabrication sensitivity. The Q=30,000 limit across designs exemplifies the breakdown of our fabrication-sensitivity model, indicating that an alternative source of error is limiting device Q.

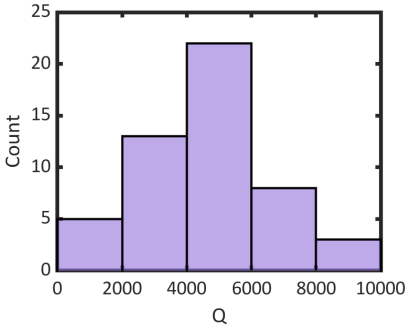

Figure S.14: Measurement results of several high-Q designs fabricated using a commercial MOCVD wafer. Plotted are the results for three different cavity designs, all with nominal Q exceeding 100,000. For all fabricated cavities, a maximum quality factor of 10,000 is observed using the MOCVD wafers.

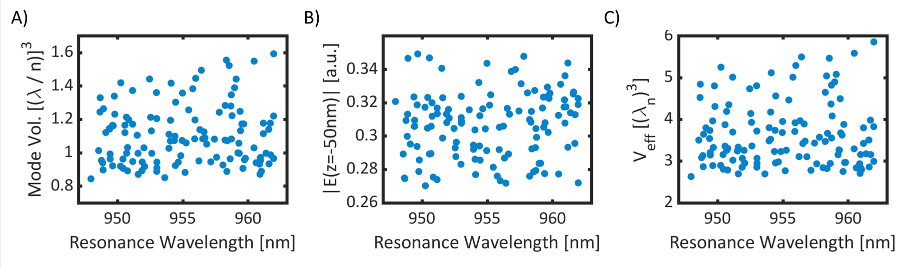

Figure S.15: A) Normalized mode volumes of the cavities from figure 2 of the main text. B) Maximum normalized field strength of the cavity field profiles at a target depth of 50 nm. C) Maximum effective mode volumes at the target depth.

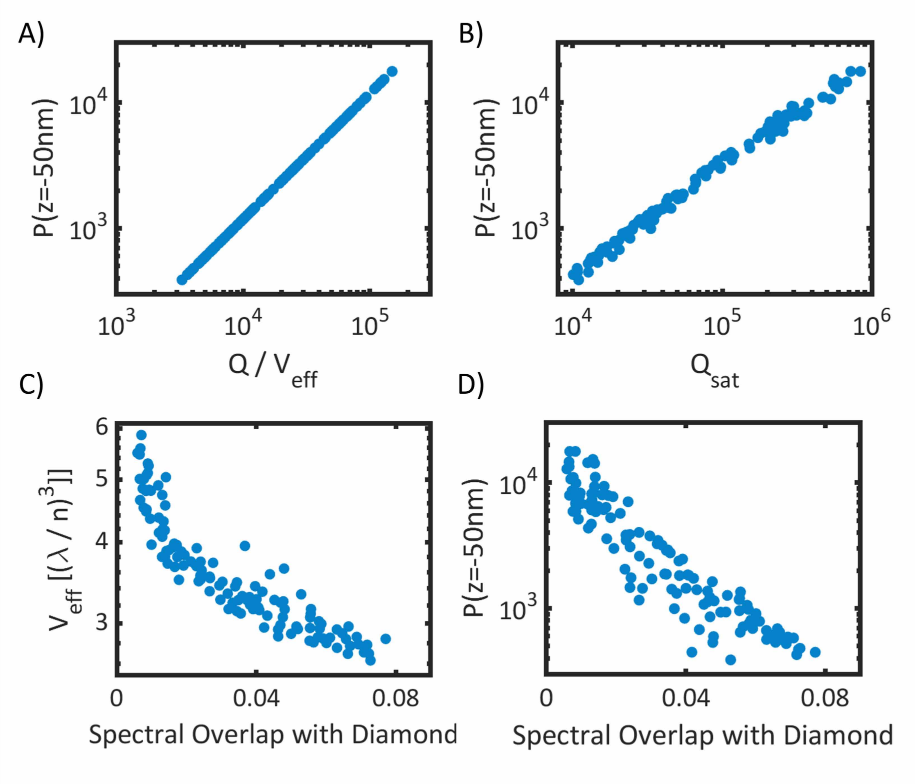

Figure S.16: Maximum Purcell enhancement for SiV^0 at a depth of 50 nm for the cavities considered in this work. Purcell is plotted as a function of A) saturated quality factor divided by the effective mode volume, and B) saturated quality factor. C) Effective mode volume and D) Purcell enhancement as a function of the spectral overlap with diamond radiative modes.

	Step 1 - Gas Stabilization	Step 2 - Etch
Time (s)	60	30
RF Power (W)	0	50
ICP Power (W)	0	500
Chamber Pressure (mTorr)	5	5
Cl_2 Flow Rate (sccm)	10	10
Ar Flow Rate	10	10
BCl_3 Flow Rate	10	10
N_2 Flow Rate	10	10

Table 1: Rapid etch recipe. The etch parameters are chosen as a baseline which achieves vertical sidewalls outside of the PhC holes with high selectivity. The etch is performed at a chuck temperature of 20°C.

	Step 1 - Gas Stabilization	Step 2 - Strike	Step 3 - Etch
Time (s)	60	3	330
RF Power (W)	0	25	25
ICP Power (W)	0	50	50
Chamber Pressure (mTorr)	2	5	2
Cl_2 Flow Rate (sccm)	1.75	1.75	1.75
Ar Flow Rate	2	2	2
N_2 Flow Rate	1	1	1

Table 2: ARDE-optimized etch recipe. The ICP and RF powers are reduced to the lowest values able to support a stable plasma at a pressure of 2 mTorr. The N_2 is set to the minimum value according to the tool mass-flow controllers (MFC), while the Cl_2 flow is adjusted to achieve vertical sidewalls within the PhC holes. Argon is set to the minimum supported by the MFC and is critical to maintaining plasma stability. The etch is performed at a chuck temperature of 20°C.
